# Ketamine Infusion for Sedation and Analgesia during Mechanical Ventilation in the ICU: A Multicenter Evaluation

**DOI:** 10.1155/2022/9853344

**Published:** 2022-11-30

**Authors:** Kathryn M. Pendleton, Laurel E. Stephenson, Nick Goeden, Anna R. Benson, Qi Wang, Salman B. Mahmood, Kelly A. Considine, Matthew E. Prekker

**Affiliations:** ^1^Division of Pulmonary, Allergy Critical Care and Sleep Medicine, University of Minnesota, Minneapolis, MN, USA; ^2^Abbott Northwestern Hospital, Minneapolis, MN, USA; ^3^University of Minnesota Medical Center, Minneapolis, MN, USA; ^4^Clinical and Translational Science Institute, University of Minnesota, Minneapolis, MN, USA; ^5^Department of Medicine, Hennepin County Medical Center, Minneapolis, MN, USA; ^6^Division of Pulmonary and Critical Care Medicine, Hennepin County Medical Center, Minneapolis, MN, USA; ^7^Department of Emergency Medicine, Hennepin County Medical Center, Minneapolis, MN, USA

## Abstract

**Methods:**

We reviewed the electronic medical record of critically ill adults who received a continuous infusion of ketamine for ≥24 hours during invasive mechanical ventilation in three hospitals over a two-year period. We captured data including ketamine indication, dose, unintended effects, and adjustments to coadministered sedatives or opioids. We analyzed these data to determine the incidence of reported unintended effects of ketamine infusion (primary outcome) and changes in exposure to coadministered sedatives or opioids during ketamine use (secondary outcome).

**Results:**

95 mechanically ventilated adults received a ketamine infusion for a median duration of 75 hours (interquartile range [IQR] 44–115) at a mean ± standard deviation (SD) infusion rate of 1.3 ± 0.5 mg/kg/hour for the first 24 hours. At least one unintended effect attributed to ketamine was documented in 24% of cases, most frequently tachycardia (6%) and sialorrhea (6%). Other sedative or opioid infusions were administered with ketamine in 76% and 92% of cases, respectively. Comparing the total amount of sedative or opioid administered in the 24 hours prior to ketamine infusion with the total amount administered during the first 24 hours on ketamine, there were no significant differences in propofol, midazolam, or dexmedetomidine exposure, but the average fentanyl exposure was higher after ketamine (2740 ± 1812 mcg) than before (1975 ± 1860 mcg) (absolute difference 766 mcg, 95% confidence interval [CI] 442 to 1089 mcg).

**Conclusions:**

In this multicenter cohort of critically ill, mechanically ventilated adults, ketamine infusion was primarily used as an adjunct to conventional sedative and opioid infusions, with noticeable but unintended effects potentially related to ketamine in nearly one-quarter of cases.

## 1. Introduction

Patients in the intensive care unit (ICU) frequently receive parenteral sedatives during mechanical ventilation to promote synchrony with the ventilator, achieve stable hemodynamics or gas exchange, and treat anxiety and pain. There is growing interest in nontraditional sedative drugs in the ICU as part of a multimodal sedato-analgesia strategy to maintain an appropriate depth of sedation as recommended by clinical guidelines [1]. Traditional sedative agents have well-described adverse effects, including toxic accumulation, hemodynamic instability, and increased risk of ICU delirium, which has been independently associated with worse patient-centered outcomes [1–3].

Ketamine is a rapidly acting, dissociative anesthetic agent that has been in clinical use since the 1960s [4]. It is a phencyclidine (PCP) derivative that interacts with multiple binding sites, including N-methyl-D-aspartate (NMDA) and non-NMDA glutamate receptors, nicotinic and muscarinic cholinergic, and monoaminergic and opioid receptors [5]. Classic ketamine anesthetic effects are best described as a dose-dependent central nervous system depression [6]. Ketamine ideally provides dissociative sedation, anterograde amnesia, and analgesia while respiratory effort and cardiovascular stability are generally preserved. Its duration of action is approximately 30–45 minutes with a half-life of 10–15 minutes, making it particularly suitable for situations in which a short-term, rapidly acting analgesic, or sedatives are needed [7]. For this reason, it has been hypothesized that ketamine may be an ideal single agent sedative for ICU patients. While these characteristics and pharmacodynamic properties have led to widespread use during procedural sedation, the use of ketamine infusions for sedation in critically ill adults has received less attention than conventional sedatives in the critical care literature. Despite this, a recent national, epidemiologic study found that use of continuous ketamine in critically ill patients receiving mechanical ventilation has been steadily increasing over the last decade suggesting clinicians are willing to use this medication despite the paucity of literature available to guide them clinically [8].

The purpose of this study was to determine the incidence of adverse events associated with ketamine used as a sedative in adult patients in the ICU who require mechanical ventilation for greater than 24 hours. We also sought to determine if use of ketamine facilitated sparing of other opiate or sedative infusions. By quantifying the frequency of adverse effects observed during exposure to ketamine infused to provide dissociative sedation, this study aims to determine the incidence of reported unintended effects of ketamine infusion (primary outcome) and changes in exposure to coadministered sedatives or opioids during ketamine use (secondary outcome).

## 2. Methods

This multicenter retrospective study of a convenience sample of critically ill adults who received an intravenous ketamine infusion took place in one of three urban hospitals: a university-based academic medical center; an urban county Level 1 Trauma Center; and a community hospital with a mixed medical and surgical ICU population. The relevant Institutional Review Boards approved the study protocol (IRB 00001930 and HSR 18–4515).

Patients were included if they were greater than 18 years of age, admitted to an ICU, received invasive mechanical ventilation for at least 24 consecutive hours, and concomitantly received ketamine for greater than 24 hours at an initial infusion rate of at least 0.5 mg/kg/hr. The minimal starting infusion rate of at least 0.5 mg/kg/hr was chosen as best available evidence supports this is above the commonly accepted dosage range used for acute pain management and thus is likely to represent dissociative dosing [9]. There were no specific clinical exclusion criteria, although patients who indicated in their medical record their desire not to participate in clinical research were excluded. Eligible patients who received ketamine during the study period of January 1, 2015 through December 31, 2017 were identified using pharmacy dispensing records. All patients who met inclusion criteria were included in the safety analysis. Patients who received ketamine for the primary indication of sedation were included in a subgroup analysis of the adequacy of sedation, as measured by target versus actual Richmond-Agitation-Sedation-Scale (RASS) values. As frontline use of ketamine as an analog sedative agent in mechanically ventilated patients has not been supported by current clinical practice guidelines [1], this agent was not routinely being used for this purpose at any of the study institutions prior to or during the study period. However, all three institutions had newly developed pharmacy protocols for use of ketamine infusions in the ICU, and the decision to use this agent was at the discretion of the responsible physicians.

Data were collected on the study cohort by manual review of the electronic health record (EHR) at each institution. This record review was carried out by trained investigators using a standardized data collection form. Parameters abstracted from the EHR to assess the safety of ketamine dosing to achieve dissociative sedation include the incidence of hypertension, tachycardia, or tachyarrhythmias defined as onset within 1 hour of beginning the ketamine infusion. Incident tachycardia was defined as an increase in heart rate of 15 or more beats/minute over baseline and incident hypertension was defined as systolic blood pressure >180 mm Hg. The EHR was reviewed for any mention of increased agitation or signs of emergence reactions within one hour of ketamine discontinuation. Patients receiving ketamine primarily for sedation, rather than for pain management, were included in a subgroup analysis to assess the efficacy of ketamine as a sedative agent. For the first 24 hours of ketamine infusion, recorded RASS scores were compared against goal RASS targets ordered by the responsible clinician and entered into the medication order. Total doses of other sedative and opioid classes of medications were compared 24 hours preketamine and 24 hours postketamine.

### 2.1. Statistical Analysis

Data are reported using mean and standard deviation, or median and interquartile range, for continuous variables while categorical variables are reported using counts and proportions. Descriptive statistics were calculated to compare the distribution of sedative and opioid dosing before and during ketamine administration; these paired data were analyzed using the Wilcoxon signed rank test and paired *t*-test, as appropriate. Analysis was performed using Stata 15 (StataCorp, College Station, TX). *P* values < 0.05 were considered statistically significant, however based on the study design inferential comparisons should be considered exploratory.

## 3. Results

**Figure 1:**
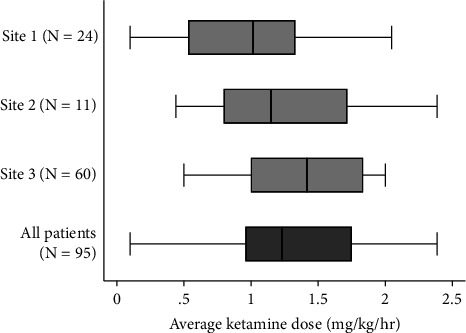
Ketamine Dose. The distribution of average ketamine dose (mg/kg/hr) in the first 24 hours of use by study site and for all patients. The vertical line (within the shaded boxes) represents the median value, the shaded boxes represent the interquartile range, and the whiskers represent adjacent values.

95 critically ill patients receiving invasive mechanical ventilation (median age 50 years [IQR 35–61], 66% male, median SOFA score 9 [IQR 7–12], concomitant extracorporeal membrane oxygenation [ECMO] in 9%) received ketamine by continuous intravenous infusion for at least 24 consecutive hours during the study period (Table 1). The median number of days from ICU admission to initiation of ketamine infusion was 2 (IQR 1–10) and the median duration of ketamine infusion was 3 days (IQR 2–5). The median total dose of ketamine administered over the first 24 hours is 30 mg/kg (IQR 23–42), which is equivalent to a median hourly dose of 1.3 mg/kg/hr (IQR 0.96–1.8). The maximal ketamine administration rate at any hourly interval in the first 24 hours was 5.2 mg/kg/hr and the maximal infusion rate was 2 mg/kg/hr in 47 patients (49%) and >2 mg/kg/hr in eight patients (8%). The distribution of ketamine dosing over the first 24 hours is shown in Figure 1.

A total of 23 patients (24%) experienced at least one adverse effect potentially related to ketamine treatment. Within one hour of ketamine initiation, 6 patients (6%) developed new or worsening tachycardia, 3 patients (3%) developed incident hypertension (2 of these 3 patients were treated with parenteral anti-hypertensive medication), and no patients developed tachyarrhythmia. During ketamine infusion, 6 patients (6%) developed sialorrhea, 5 patients (5%) developed an acute change in their neurologic examination prompting brain imaging or neurology consultation, and 5 patients (5%) were observed to have emergence phenomena after ketamine infusion was stopped. Data regarding incidence of adverse events stratified by ketamine dose is provided in Table 2. Cardiac arrest occurred during the hospitalizations of 4 study patients (4%), three of whom were receiving ketamine at the time of the cardiac arrest. The events were adjudicated by two investigators (KMP and MEP) according to the Adverse Drug Reaction Probability Scale (Naranjo) [10]. Following adjudication, ketamine could have possibly contributed to the cardiac arrest in 1 of the 4 patients. The one case of cardiac arrest potentially attributable to ketamine involved a patient admitted with acute hypoxic respiratory failure related to pancreatitis and acute respiratory distress syndrome. On ICU day 5, while receiving mechanical ventilation, ketamine infusion was started owing to high sedation needs with breakthrough agitation. On ICU day 8, 3 days after starting ketamine, the patient suffered a cardiac arrest after a period of bradycardia. Return of spontaneous circulation was achieved after approximately 2 minutes of CPR. A mixed respiratory and metabolic acidosis was noted immediately following the arrest (pH 6.9). In the hour leading up to the cardiac arrest, the ketamine was increased from 120 to 150 mg/hr, midazolam infusion was increased from 6 to 10 mg/hr, and the patient received an IV bolus of fentanyl 50 mcg. Documentation indicated a possible ketamine administration error of unknown duration: the ketamine was being delivered via an infusion pump set to mL/hr rather than mg/hr, resulting in approximately double the expected dose of ketamine to be administered per hour. Please see Supplementary Table 1 for additional clinical details on these cases.

Physicians documented various rationale for using ketamine in these mechanically ventilated adults: 35% aimed to decrease exposure to other sedative-analgesic drugs, 33% sought a neutral hemodynamic profile, and 21% sought a unique mechanism of action as conventional sedatives failed to achieve sedation goals, among other reasons. Ketamine was most often chosen as a replacement for propofol with common reasons for change being hypertriglyceridemia limiting propofol use or presence of a shock state requiring significant doses of multiple vasopressors. In many instances, ketamine was added to existing sedative regimens without cessation of other agents due to inadequate depth of sedation on the preketamine regimen, usually in the setting of refractory seizures or need for neuromuscular blockade initiation.

In 92 of 95 patients (97%), ketamine was administered concomitantly with at least 1 other sedato-analgesic infusion. Specifically, these coadministered drugs included opioids (92%), propofol (48%), benzodiazepines (47%), or dexmedetomidine (12%). An opioid drug, without other sedatives, was paired with ketamine in 20 instances (21%), an opioid and a sedative were used with ketamine in 67 instances (71%), and a sedative drug alone was paired with ketamine in 5 instances (5%).

**Figure 2:**
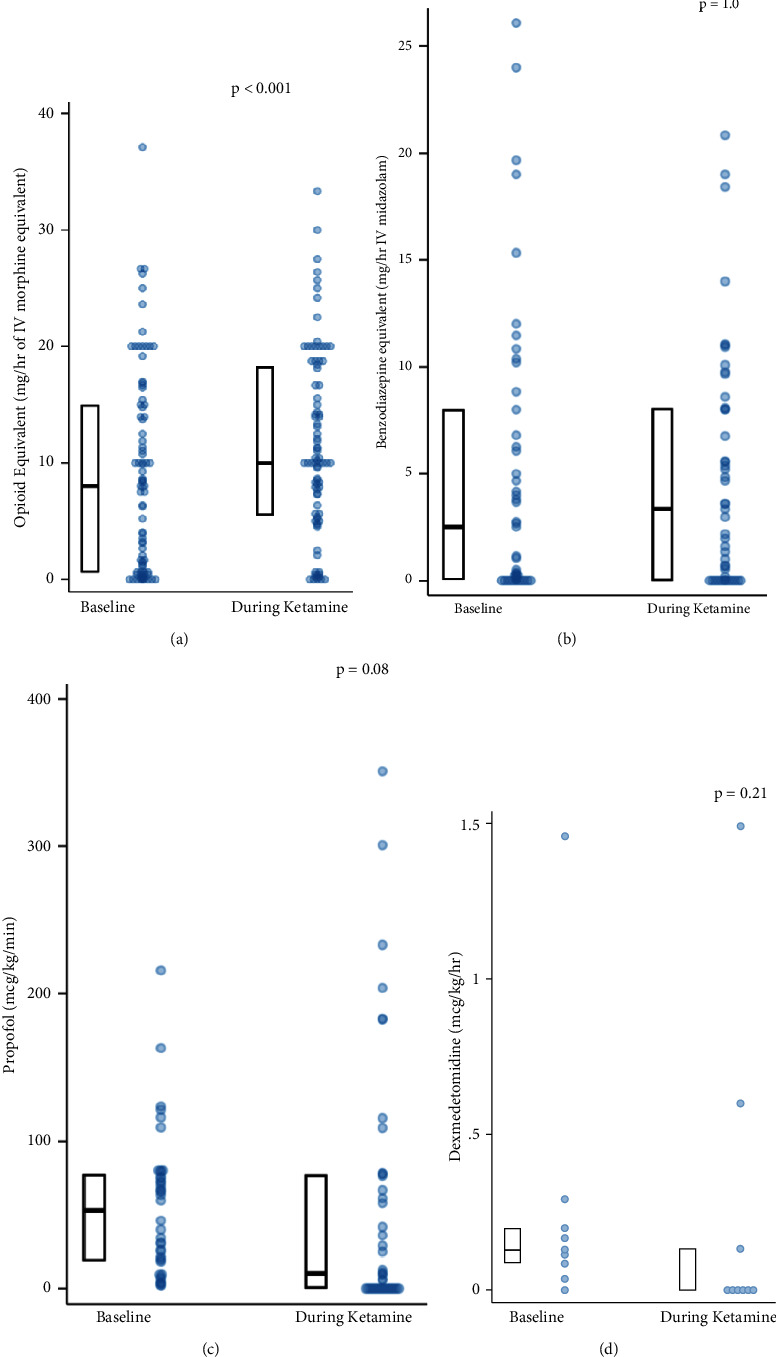
Doses of select sedative and analgesic drugs administered at baseline and during ketamine use. The four panels show opioids (a), benzodiazepines (b), propofol (c), and dexmedetomidine (d). The Wilcoxon signed rank test was used to compare median dose values of these drugs at baseline and during ketamine and generated *P* values are shown.

As compared to the 24 hours prior to ketamine initiation, the median total dose of opioid analgesia (expressed morphine equivalents [mg/hr]) was higher during ketamine infusion (Figure 2). Fentanyl was the most frequently coadministered opioid and fentanyl exposure was higher, on average, during ketamine (2740 ± 1812 mcg) than the 24 hours prior to ketamine initiation (1975 ± 1860 mcg) (absolute difference 766 mcg, 95% CI 442 to 1089 mcg). In contrast, the median exposure to propofol and dexmedetomidine was less after ketamine initiation, on average (Figure 2). For the patients coadministered with benzodiazepine infusion, the exposure to benzodiazepine was similar before and after the start of ketamine infusion. However, in the subgroup of patients with status epilepticus (*N* = 10), there was a lower median benzodiazepine dose after ketamine initiation (8 mg/hr midazolam equivalent [IQR 0–43]) as compared to the 24 hours prior to ketamine initiation (21 mg/hr [IQR 1–56]) (*P*=0.09). Considering the depth of sedation among all patients, the RASS score was lower in the 24 hours after ketamine initiation, on average, as compared to the RASS score in the 24 hours preceding ketamine (−2.7 vs. −2.0, respectively, absolute difference 0.7, 95% CI 0.4–1.0, *P* < 0.001).

## 4. Discussion

Prioritization of sedation minimization, daily sedation interruption, and the use of nonbenzodiazepine-based sedatives has led to renewed interest in ketamine. Studies have suggested that lower plasma concentrations of ketamine may provide the so-called sweet spot to achieve adequate sedation, or dissociation, while minimizing adverse psychomimetic effects [11, 12]. For this reason, initiation of ketamine infusions has been theorized to be beneficial either in isolation or adjunct medication in ICU sedation. Whereas ketamine has historically been popular for the dissociative state of anesthesia it provides without adversely affecting hemodynamic status, concerns remain regarding side effects of hallucination and delirium, particularly as they relate to critically ill patients. In this study, unintended clinical effects were documented in nearly one quarter of cases. The most common were new tachycardia and sialorrhea, but more concerning effects were also observed including a change in neurologic status prompting cranial imaging (5%) and emergence phenomena when ketamine was stopped (5%).

The study period encompasses the early experiences of these ICU clinicians with continuous ketamine infusion for analgosedation during mechanical ventilation. ICU ketamine protocols were newly implemented in the 3 participating institutions in 2015. We did not find that initiation of ketamine had a sparing effect on other sedative and analgesic infusions within 24 hours. We speculate that this could be attributed to nursing staff and providers being relatively unfamiliar with this drug and with the appearance of a patient under dissociative sedation in general, leading to overall lack of aggressive titration and dose adjustments. While some studies have found that ketamine use may be associated with an opiate sparing effect, we did not find either to be the case in our study [13, 14]. As “inadequate sedation” and “hemodynamic reasons” were oft-cited indications for switching to ketamine infusion, we hypothesize that the lack of difference observed in our study is more reflective of patient condition than properties of ketamine.

Our observations around unintended effects and coadministration of other analgosedative infusions during ketamine use generally support similar single-center studies already published [15, 16]. Existing studies concerning ketamine in this context have demonstrated conflicting results in terms of opioid and other sedative sparing effects as well as the development of delirium. Larger prospective trials are forthcoming [17, 18] and will be helpful in better establishing efficacy of ketamine as an ICU sedative agent; however, the presence of a few smaller studies suggests that ICU practitioners are comfortable using ketamine, even in the absence of more robust data.

This study has several strengths. First, generalizability of these results is improved by the multicenter design; the three study hospitals are complementary referral centers in our region, specializing in quaternary care and complex organ failures (university academic medical center), critical care for patients with polytrauma or serious burns (county Level 1 Trauma Center), and complex cardiovascular illnesses (community hospital and hub of regional referral network). Two of these hospitals provide adult extracorporeal membrane oxygenation (ECMO) and have used ketamine in that setting. Second, collaboration between physician investigators and critical care pharmacists in nuanced data abstraction across hospitals led to a robust look at pre and post ketamine dosing and blinded adjudication of adverse effects.

These results should be interpreted in the context of several limitations. First, the main limitation of our study is its retrospective design and its inherent issues of missing data and observer bias. As continuous ketamine infusions are not currently routinely used for ICU sedation, this study can only provide a descriptive report on our experience with dosing, efficacy, and recorded adverse effects. We did not collect detailed hemodynamic data at baseline or during ketamine infusion in this cohort, so we are unable to quantify changes in the distribution of blood pressure or heart rate before and after ketamine administration. Next, one of the frequent indications for starting ketamine was provider preference, which could have introduced selection bias. Twenty-five percent of patients in our cohort were administered ketamine continuously for more than 5 days, however these data are inadequate to inform the long-term safety of ketamine infusion as our follow-up period ended at hospital discharge. Finally, we had hoped to be able to comment on any associations between ketamine and the development and prevalence of ICU delirium but found that delirium screening recording in the EHR was too erratic for meaningful interpretation. Future studies investigating the propensity of ketamine to increase or decrease risk of ICU delirium will be paramount to deciding whether ketamine can be used routinely for ICU sedation.

## 5. Conclusion

In this multicenter cohort of critically ill mechanically ventilated adults, ketamine infusion was primarily used as an adjunct to conventional sedative and opioid infusions, with noticeable but unintended effects potentially related to ketamine in nearly one-quarter of cases.

## Figures and Tables

**Table 1:** Baseline patient characteristics (overall and stratified by ketamine dose).

Variable	Ketamine dose group			All patients (*N* = 95)
	0.1–0.99 mg/kg/hr (*N* = 27)	1.0–1.49 mg/kg/hr (*N* = 32)	1.5–2.4 mg/kg/hr (*N* = 36)	
Age, y	50 (39–62)	51 (32–61)	47 (33–59)	50 (35–61)
Male sex	15 (56%)	22 (69%)	26 (72%)	63 (66%)
BMI, kg/m^2^	26 (23–32)	25 (23–32)	28 (24–32)	26 (23–32)
SOFA score^*∗*^	10 (8–14)	10 (6–12)	8 (6–11)	9 (7–12)
Reason for ICU admission				
Respiratory failure	7/23 (30%)	7/23 (30%)	9/23 (39%)	23 (24%)
Status epilepticus	4/13 (31%)	6/13 (46%)	3/13 (23%)	13 (14%)
Trauma including burns	4/12 (33%)	6/12 (50%)	2/12 (17%)	12 (13%)
Septic shock	3/12 (25%)	4/12 (33%)	5/12 (42%)	12 (13%)
Cardiovascular disease	2/11 (18%)	4/11 (36%)	5/11 (45%)	11 (12%)
Overdose	1/7 (14%)	1/7 (14%)	5/7 (71%)	7 (7%)
Asthma/COPD	1/6 (17%)	2/6 (33%)	3/6 (50%)	6 (6%)
Other^*∗∗*^	5/11 (45%)	2/11 (18%)	4/11 (36%)	11 (12%)

Values are given as median (interquartile range) or count (percentage). ^*∗*^SOFA score is missing for 13 patients (*N* = 82). ^*∗∗*^other reason for ICU admission: gastrointestinal hemorrhage (*N* = 4), procedure (*N* = 3), diabetic ketoacidosis (*N* = 1), severe pancreatitis (*N* = 1), environmental hypothermia (*N* = 1), and ischemic stroke (*N* = 1). BMI, body mass index; SOFA, sepsis related organ failure assessment score; ICU, intensive care unit.

**Table 2:** Potential adverse effects of ketamine and patient outcomes (overall and stratified by ketamine dose).

Variable	Ketamine dose group			All patients (*N* = 95)
	0.1–0.99 mg/kg/hr (*N* = 27)	1.0–1.49 mg/kg/hr (*N* = 32)	1.5–2.4 mg/kg/hr (*N* = 36)	
Potential adverse effects of clinical significance, any	4 (29%)	5 (36%)	5 (36%)	14 (100%)
Hypertension	1	0	2	3 (21%)
Acute neurologic change	3	1	1	5 (36%)
Emergence phenomena	0	3	2	5 (36%)
Cardiac arrest^*∗*^	1	2	1	4 (4%)
Duration of mechanical ventilation, days	9 (7–19)	10 (6–16)	9 (3–15)	9 (5–16)
ICU length of stay, days	14 (9–28)	15 (9–24)	11 (4–23)	14 (7–23)
Hospital mortality	13 (48%)	6 (19%)	11 (31%)	30 (32%)

Values are given as median (interquartile range) or count (percentage). ^*∗*^Cardiac arrest was defined by its occurrence at any time during the index hospitalization after exposure to ketamine. After adjudication, ketamine could have possibly contributed to the cardiac arrest in 1 of the 4 patients, and that patient was in the 1.0–1.49 mg/kg/hr dose group.

## Data Availability

Access to data is restricted due to patient confidentiality.

## Abbreviations

ICU: Intensive care unit
PCP: Phencyclidine
NMDA: N-Methyl-D-aspartate
RASS: Richmond-agitation-sedation-scale
EHR: Electronic health record
ECMO: Extracorporeal membrane oxygenation

## Supplementary Materials

Supplementary Materials Supplemental table 1 shows the clinical details of in-hospital cardiac arrest experienced by 4 patients who received ketamine infusion.
