# Supplementary material for: Ketamine Infusion for Sedation and Analgesia during Mechanical Ventilation in the ICU: A Multicenter Evaluation
Source: Crit Care Res Pract. 2022 Nov 30;2022:9853344. doi: 10.1155/2022/9853344 (PMC9729046; doi:10.1155/2022/9853344)
Supplement: Supplementary Materials — Supplemental table 1 shows the clinical details of in-hospital cardiac arrest experienced by 4 patients who received ketamine infusion. [file 9853344.f1.docx]

## **Supplemental Table 1.** Clinical details of in-hospital cardiac arrest experienced by 4 patients who received ketamine infusion. The probability of an event being related to an adverse drug reaction was adjudicated according to the Adverse Drug Reaction Probability Scale (Naranjo).

| Patient | Related to Ketamine | Case Description |
| --- | --- | --- |
| 1 | Doubtful | Young adult male admitted to the ICU with polytrauma after a motor vehicle crash. His injuries included thermal burns to his face covering 20% of his total body surface area. Ketamine infusion (dose range 1-2 mg/kg/hr) was used for sedation during mechanical ventilation for the entirety of his 19-day hospitalization. On day #19 while receiving invasive mechanical ventilation through tracheostomy tube, he became difficult to ventilate and suffered an asystolic cardiac arrest. Emergent evaluation revealed mucoid concretions obstructing the distal tracheostomy tube; despite removing the obstruction and re-establishing ventilation, he died. |
| 2 | Doubtful | Elderly woman who was admitted to the general medical floor with dyspnea thought due to COPD and/or CHF. On hospital day #2 she suffered a cardiac arrest due to ventricular tachycardia. She underwent endotracheal intubation during the code and received ketamine infusion during the initial 48 hours in the ICU, owing to persistent hypotension with propofol sedation. After ketamine was stopped, she suffered two additional pulseless arrests also due to ventricular arrythmias thought due to non-ST elevation myocardial infarction (type II). She was discharged on hospice care. |
| 3 | Doubtful | Middle-age male with a history of paraplegia and sacral decubitus ulcers who was admitted to the ICU with septic shock and respiratory failure related to infected sacral ulcer. He received ketamine sedation (dose range 0.5-2 mg/kg/hr) during mechanical ventilation. During the initial 72 hours, his circulatory shock worsened, and he required 4 vasopressors. In the setting of refractory hypotension on day #3, he suffered a bradycardic-PEA cardiac arrest. Resuscitation was ultimately unsuccessful, and he died. |
| 4 | Possible | Middle-age female was admitted to the ICU with acute respiratory failure due to ARDS in the setting of severe pancreatitis. On ICU day #5, while receiving mechanical ventilation, ketamine infusion was started owing to high sedation needs with breakthrough agitation. On ICU day #8, 3 days after starting ketamine, the patient suffered a cardiac arrest after a period of bradycardia. Return of spontaneous circulation was achieved after approximately 2 minutes of CPR. She had a mixed respiratory and metabolic acidosis immediately following the arrest (pH 6.9). In the hour leading up to the cardiac arrest, the ketamine was increased from 120 to 150 mg/hr, midazolam infusion was increased from 6 to 10 mg/hr, and she received an IV bolus of fentanyl 50 mcg. Documentation indicated a possible ketamine administration error of unknown duration: the ketamine was being delivered via an infusion pump set to mL/hr rather than mg/hr, resulting in approximately double the expected dose of ketamine to be administered per hour. |
